# Surgical Outcomes of Trabeculectomy in Uveitic Glaucoma: A Long-Term, Single-Center, Retrospective Case-Control Study

**DOI:** 10.1155/2021/5550776

**Published:** 2021-05-21

**Authors:** Rina Kanaya, Riki Kijima, Yasuhiro Shinmei, Akihiro Shinkai, Takeshi Ohguchi, Kenichi Namba, Shinki Chin, Susumu Ishida

**Affiliations:** Department of Ophthalmology, Faculty of Medicine and Graduate School of Medicine, Hokkaido University, Sapporo, Japan

## Abstract

**Purpose:**

To evaluate the long-term outcomes of trabeculectomy with mitomycin C (MMC-TLE) in patients with uveitic glaucoma (UG). *Patients and Methods.* This was a retrospective, nonrandomized case series study. MMC-TLE was performed on 50 eyes with UG between February 2001 and January 2015 at Hokkaido University Hospital. Age- and sex-matched patients with primary open angle glaucoma (POAG) who underwent MMC-TLE were matched by age and sex and enrolled as controls. Surgical success was defined as an intraocular pressure (IOP) less than 18 or 15 mmHg. The Kaplan–Meier survival curves for surgical failure were analyzed.

**Results:**

The mean preoperative IOP in UG and POAG was 27.6 ± 10.6 and 18.0 ± 4.5 mmHg, respectively. After the surgery, the mean IOP in UG and POAG was reduced to 11.7 ± 4.2 and 12.2 ± 3.8 mmHg at 12 months, 11.9 ± 7.0 and 12.1 ± 3.1 mmHg at 36 months, and 13.0 ± 5.2 and 10.6 ± 1.2 mmHg at 120 months, respectively. The success rates (IOP <18 mmHg, IOP reduction >20%) in UG and POAG were 91.7% and 88.0% at 12 months, 82.2% and 75.6% at 36 months, and 66.5% and 61.8% at 120 months, respectively. The success rates (IOP <15 mmHg) in UG and POAG were 64.0% and 58.0% at 12 months, 55.1% and 45.5% at 36 months, and 47.9% and 37.8% at 120 months, respectively. There was no significant difference in the success rate between UG and POAG at 120 months after surgery by either definition of surgical success.

**Conclusions:**

MMC-TLE effectively reduced IOP in both UG and POAG. There was no significant difference in the success rate between UG and POAG. Following sufficient inflammation suppression, surgical outcomes of UG may be comparable with those of POAG.

## 1. Introduction

Secondary glaucoma is a common problem in eyes with uveitis and can cause blindness if not treated. Uveitis may lead to increased intraocular pressure (IOP), and up to 20% of patients with uveitis will develop glaucoma [1, 2]. Increased eye pressure in uveitis can result from the inflammation itself or from the steroids used to treat it. Topical and systemic corticosteroids are useful in treating uveitis and uveitic glaucoma, but approximately 30% of eyes with uveitic glaucoma (UG) require surgical intervention to control IOP [3].

The increase in IOP in UG is caused by a breakdown of the blood-ocular barrier, followed by the influx of proteins, immunocompetent cells, cytokines, and chemokines, which obstruct the trabecular meshwork (TM), directly damaging the tissue and altering the function of the TM [4, 5].

We previously assessed the outcome of a modified 360-degree suture trabeculotomy for UG [6, 7]. The surgical procedure reduced resistance of the TM, but patients with a markedly affected visual field (defined as stage 5 or 6 in the modified Aulhorn–Greve classification) were excluded because it may lead to the loss of fixation by transiently increased IOP after trabeculotomy.

Trabeculectomy with mitomycin C (MMC-TLE), a filtering surgery that relies upon a bleb to filter out fluid from the anterior chamber to control IOP, remains the gold standard treatment for UG and primary open angle glaucoma (POAG) with a markedly affected visual field [8–16]. Recently, Ex-PRESS^®^ shunts [17] and XEN^®^ gel stents [18] have been developed to improve filtering surgery, but their surgical outcomes for UG are not yet known. While some glaucoma drainage device surgeries (Baevelt, Molteno, and Almed) have been performed for UG, the outcomes were not superior to those of MMC-TLE [19, 20].

Previous studies compared the outcomes of MMC-TLE between patients with POAG and patients with UG [9, 10, 12]; however, the evaluations were incomplete because of the wide range of uveitis subtypes and inconsistent patient backgrounds.

The purpose of this study was to evaluate the outcome of MMC-TLE in patients with UG and to define risk factors for surgical failure, excluding the effects of age and sex, which are known risk factors.

## 2. Patients and Methods

This study was a retrospective, nonrandomized case series at a single ophthalmological institution. A consecutive series of 50 patients with uveitic glaucoma underwent MMC-TLE at Hokkaido University Hospital between February 2001 and January 2015. Eight patients with combined cataract surgery were included. Uveitic glaucoma was defined as glaucoma with previously diagnosed uveitis or uveitis diagnosed at the time of presentation in which a high IOP of greater than 21 mmHg on consecutive visits was observed with glaucomatous optic nerve head changes. One eye was randomly selected from each patient by the randomization tool (Microsoft Excel 2010) if both eyes met the inclusion criteria. Another group of 50 eyes in 50 age- and sex-matched patients with POAG who underwent MMC-TLE during the same period were used as controls. In all pseudophakic eyes, cataract surgery was performed over 3 months before MMC-TLE (16 eyes in UG and 8 eyes in POAG). In all eyes with UG, inflammation was under control prior to the surgery with topical and/or systemic oral prednisolone. During periods of intense inflammation, anti-inflammatory treatment is prioritized over surgery for at least several weeks. Furthermore, some patients with Behçet's disease were treated using infliximab. Surgery was performed for patients who were unable to undergo modified 360-degree suture trabeculotomy because of their markedly affected visual fields. This study complied with the Health Insurance Portability and Accountability Act and adhered to the tenets of the Declaration of Helsinki. It was reviewed and approved by the institutional review board of Hokkaido University Hospital for clinical research and registered in UMIN-CTR (ID: UMIN000020879). Written informed consent was received from all patients enrolled in this study.

Surgery was performed by 3 glaucoma surgeons (Y. S., C. S., or T. O.) under local peribulbar anesthesia in all patients. Following placement of a corneal traction suture using 6-0 polypropylene sutures, the surgeon dissected a fornix-based conjunctival flap and then created a one-half-thickness scleral flap (4 × 4 mm). Subsequently, MMC was applied to the sclera over the proposed scleral flap site with sponges containing 0.04% MMC solution positioned and maintained in the subconjunctival space for 3 min. After removal of the sponges, the area was irrigated with 100 ml of physiologic saline. Peripheral iridectomy was performed during trabeculectomy, with a block of clear cornea and trabecular meshwork tissue at the edge of the corneoscleral bed removed during the procedure. Using 4 to 5 monofilament 10-0 nylon sutures, the scleral flap was sutured. The conjunctiva was closed with 9-0 virgin silk sutures and 10-0 nylon block sutures as previously reported [21].

In combined cataract and glaucoma surgery, phacoemulsification was performed and a foldable 6.0 mm AcrySof ® IQ IOL (SN60WF; Alcon Laboratories, Fort Worth, TX) was implanted through another 2.4 mm clear corneal incision, followed by the MMC-TLE procedure. Postoperative treatment comprised topical administration of combined antibiotic and steroid medication for 3 months. The patient underwent laser suture lysis when postoperative IOP exceeded 10 mmHg or the bleb was poorly formed. In UG patients, topical steroid was continued after 3 months as needed. Treatment for uveitis continued postoperatively as well as preoperatively including systemic oral prednisolone, immunosuppressive agents, or infliximab.

Within 60 days prior to surgery, all enrolled patients underwent baseline ophthalmic examinations as follows: history of glaucoma and medication use; best-corrected visual acuity, IOP and visual field measurements (30-2 Humphrey field analyzer; Humphrey Instruments, Munich, Germany); and slit-lamp biomicroscopic, gonioscopic, and funduscopic observations. IOP was measured using an applanation tonometer. The preoperative IOP was measured without systemic antiglaucoma drugs such as hypertonic solutions or carbonic anhydrase inhibitors. Postoperative examinations were performed during the follow-up visits at 1, 6, 12, 18, 24, 30, 36, 42, 48, 54, 60, 66, 72, 78, 84, 90, 96, 102, 108, 114, and 120 months after surgery. The surgery was considered a success if (A) the IOP was below 18 mmHg and at least 20% reduction from baseline or (B) IOP was below 15 mmHg with a similar or lower dosage of antiglaucoma medications. IOP< 5 mmHg at any postoperative visit or additional IOP-reducing procedures including bleb needling were considered to be indications of failure. According to the previous study, a postoperative increase in inflammation was defined as anterior-chamber cells that were graded as ≥2+ at any examination from 2 to 12 months after trabeculectomy [12]. Statistical analysis was performed using Microsoft Excel 2010 and BellCurve for Excel (Social Survey Research Information Co., Ltd., Tokyo, Japan). Means were compared using Student's *t*-test between two independent groups. Univariate analysis between two categorical variables was performed using Fisher's exact test. A Kaplan–Meier lifetable was used to calculate the success rate of postoperative IOP control. Statistical comparisons of the success rate between UG and POAG groups were carried out using the log-rank test. Multivariate logistic regression analysis was used to analyze the independent predictors that most influenced a dependent variable. Values are expressed as the mean ± standard deviation. Differences were considered significant at *P* < 0.05.

## 3. Results

### 3.1. Baseline Characteristics

A total of 100 patients were included for analysis in both groups (UG and POAG). Their baseline characteristics are provided in Table 1. Sex, age, and rate of combined cataract surgery were matched between groups. In total, 21 males and 29 females were enrolled in each group, and the mean age of patients in the UG and POAG groups was 62.4 ± 12.9 years and 65.0 ± 9.4 years, respectively (*P*=0.28, Student's *t*-test). Eight patients (16%) underwent MMC-TLE combined with cataract surgery in each group. Sixteen patients (32%) in UG and 8 (16%) in POAG previously underwent cataract surgery (*P*=0.10, Fisher's exact test). Two patients (4%) in UG and one (2%) in POAG previously underwent modified 360-degree suture trabeculotomy (*P*=1.00, Fisher's exact test). The other 97 patients had no history of glaucoma surgery or laser treatment prior to the surgical interventions.

The preoperative IOP values in UG (27.6 ± 10.6 mmHg) were significantly higher than those in POAG (18.0 ± 4.5 mmHg) (*P* < 0.001, Student's *t*-test). There was no significant difference in the number of preoperative topical antiglaucoma medications (2.9 ± 0.7 in UG, 3.0 ± 0.4 in POAG. *P*=0.51, Student's *t*-test). The number of patients taking carbonic anhydrase inhibitors preoperatively was significantly higher in UG than in POAG (*n* = 30 (60%) in UG and *n* = 8 (16%) in POAG. *P* < 0.001, Fisher's exact test). There was no significant difference in the preoperative MD values of HFA 30-2 SITA standard between UG (-16.3 ± 8.3 dB) and POAG (-19.4 ± 6.5 dB) (*P*=0.14, Student's *t*-test). The postoperative follow-up periods were 63.5 ± 30.7 months in UG and 75.8 ± 28.9 months in POAG (*P*=0.08, Student's *t*-test).

The phenotypic diagnoses in the UG group are shown in Table 2. They included sarcoidosis (*n* = 17, 34%), Vogt–Koyanagi–Harada disease (*n* = 4, 8%), Behçet's disease (*n* = 4, 8%), varicella zoster virus-associated uveitis (*n* = 2, 4%), Fuchs' heterochromic iridocyclitis (*n* = 1, 2%), cytomegalovirus-associated uveitis (*n* = 1, 2%), and unknown uveitis (*n* = 21, 42%).

### 3.2. Intraocular Pressure and Number of Antiglaucoma Medications

**Figure 1:**
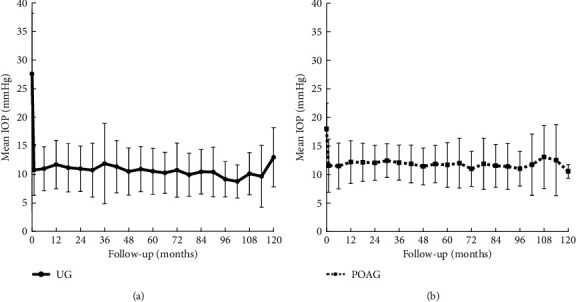
Mean preoperative and postoperative IOP after MMC-TLE in the UG and POAG groups. (a). UG. (b). POAG. The decrease in the median IOP was significant at all time points compared with the baseline values in both groups (*P* < 0.05, by Student's *t*-test). The error bar whiskers represent the standard deviation.

The mean preoperative and postoperative IOP are shown in Figure 1. The mean preoperative IOP was 27.6 ± 10.6 mmHg in UG and 18.0 ± 4.5 mmHg in POAG, and the mean postoperative IOP was 11.7 ± 4.2 mmHg and 12.2 ± 3.8 mmHg (12 months), 11.9 ± 7.0 mmHg and 12.1 ± 3.1 mmHg (36 months), 10.8 ± 4.8 mmHg and 11.0 ± 3.1 mmHg (72 months), and 13.0 ± 5.2 mmHg and 10.6 ± 1.2 mmHg (120 months) in UG and POAG, respectively. There were significant differences between the preoperative and postoperative IOP at all follow-up times (*P* < 0.01). The mean postoperative IOP values and numbers of antiglaucoma medications in UG and POAG groups every 6 months are summarized in Table 3. Although there was no significant difference in postoperative IOP between the two groups during the entire observation period, except for 30 months after surgery, the number of antiglaucoma medications was significantly lower in the UG group than in the POAG group between 6 and 36 months and between 102 and 120 months after surgery (Table 3^*∗*^*P* < 0.05,^*∗∗*^*P* < 0.01, Student's *t*-test).

### 3.3. Surgical Success

**Figure 2:**
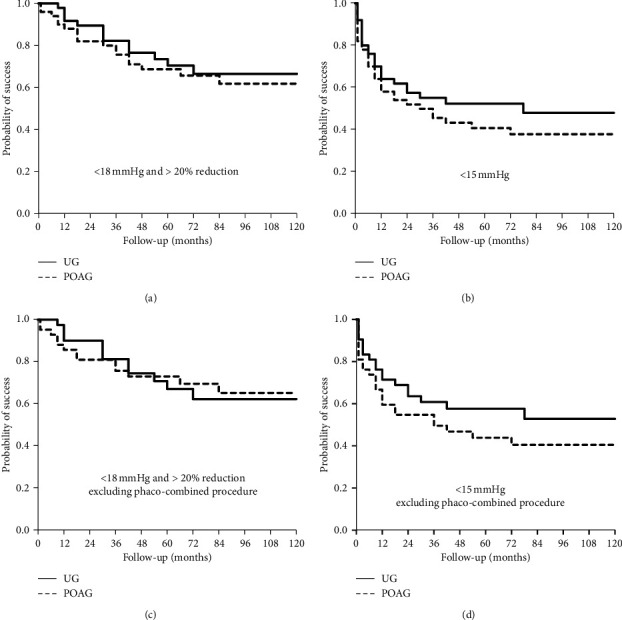
Kaplan–Meier survival curves for the success criteria comparing UG and POAG. (a) and (b) contain all 100 eyes. (c) and (d) show the results excluding phaco-combined procedure (84 eyes). Criterion: (a) and (c), IOP <18 mmHg and >20% reduction from baseline; (b) and (d), IOP <15 mmHg. There was no significant difference between UG and POAG in each criterion (*P*=0.57, 0.33, 0.99, and 0.26, respectively).

Kaplan–Meier curves for both success rates at <18 mmHg and >20% reduction from baseline (A, C) and <15 mmHg (B, D) in the UG and POAG groups are shown in Figure 2. Figures 2(a) and 2(b) include all 100 eyes, and Figures 2(c) and 2(d) include 84 eyes that underwent MMC-TLE solo (excluding phaco-combined procedure). The success rate (IOP <18 mmHg and >20% reduction from baseline) was 91.7%, 82.2%, 66.5%, and 66.5% in the UG group, and 88.0%, 75.6%, 65.7%, and 61.8% in the POAG group at 12, 36, 72, and 120 months, respectively, by Kaplan–Meier survival analysis (Figure 2(a)). The other success rate (IOP <15 mmHg) was 64.0%, 55.1%, 52.3%, and 47.9% in the UG group, and 58.0%, 45.5%, 37.8% and 37.8% in the POAG group at 12, 36, 72, and 120 months, respectively, by Kaplan–Meier survival analysis (Figure 2(b)).

Furthermore, we consider that MMC-TLE combined cataract surgery could cause more serious inflammation in UG; we excluded the result of combined surgery and performed Kaplan–Meier survival analysis. The success rate excluding phaco-combined procedure (IOP<18 mmHg and >20% reduction from baseline) was 90.0%, 81.3%, 62.3%, and 62.3% in the UG group, and 85.7%, 75.7%, 69.6%, and 65.2% in the POAG group at 12, 36, 72, and 120 months, respectively, by Kaplan–Meier survival analysis (Figure 2(c)). The other success rate excluding phaco-combined procedure (IOP<15 mmHg) was 71.4%, 60.8%, 57.6%, and 52.8% in the UG group, and 59.5%, 49.6%, 40.5%, and 40.5% in the POAG group at 12, 36, 72, and 120 months, respectively, by Kaplan–Meier survival analysis (Figure 2(d)).

There was no significant difference between UG and POAG in surgical success (A: *P*=0.57 (<18 mmHg and >20% reduction from baseline), B: *P*=0.33 (<15 mmHg), C: *P*=0.99 (<18 mmHg and >20% reduction from baseline, excluding phaco-combined procedure), D: *P*=0.26 (<15 mmHg, excluding phaco-combined procedure), log-rank test).

### 3.4. Postoperative Complications and Additional Surgeries

In our study, there were no serious intraoperative or postoperative complications that led to a loss of light perception. The postoperative complications and additional surgeries for IOP reduction or cataract progression are listed in Table 4. Only additional cataract surgery within 12 months after MMC-TLE was counted.

Early postoperative complications included aqueous leakage (4 eyes in UG, 10 eyes in POAG), shallow anterior chamber (3 eyes in UG, 5 eyes in POAG), choroidal detachment (7 eyes in UG, 2 eyes in POAG), and hyphema (7 eyes in UG, 2 eyes in POAG). Postoperative exacerbation of ocular inflammation was noted in 6 eyes of UG patients but not in POAG patients (*P*=0.03, Fisher's exact test). These 6 patients with postoperative exacerbation of ocular inflammation were diagnosed with unknown uveitis or sarcoidosis and their ocular inflammation was well controlled by topical steroid at least one year before MMC-TLE. Two of these 6 patients required cataract surgery within 12 months after MMC-TLE. Although there was no significant difference, aqueous leakage was less common in UG than in POAG (8.0%, 20%, *P*=0.15, Fisher's exact test). Choroidal detachment and hyphema were more common in UG than in POAG, but there was no significant difference.

Additional glaucoma surgeries consisted of MMC-TLE in another quadrant or Ahmed valve implant in 8 eyes in UG (16%) and 4 eyes in POAG (8%) during the follow-up period (120 months). In UG, cataract progression was noted in 5 of 34 phakic eyes (14.7%), which required cataract surgery within 12 months after MMC-TLE. In POAG, additional cataract surgery was performed for 2 of 42 phakic eyes (4.8%). There was no significant difference in the rate of additional surgery between UG and POAG (glaucoma surgery: *P*=0.36 and cataract surgery: *P*=0.14).

### 3.5. Risk Factors for Surgical Failure in UG

Based on multivariate regression analysis at 36 months after surgery (Table 5), postoperative exacerbation of inflammation was significantly correlated with surgical failure (odds ratio 74.4, 95% CI 1.83–3029.03, *P*=0.02). Additional cataract surgery after MMC-TLE was not a significant risk factor. Other baseline factors, including age, sex, granulomatous uveitis, panuveitis, combined cataract surgery, pseudophekic eye, and preoperative IOP, were evaluated but were not significant.

On the other hand, on multivariate regression analysis at 120 months after surgery (Table 6), only granulomatous uveitis was significantly inversely correlated with surgical failure (odds ratio 0.14, 95% CI 0.02–0.92, *P*=0.14).

## 4. Discussion

The aim of the present study was to investigate whether surgical outcomes of MMC-TLE differ between UG and POAG, and to clarify the risk factors for surgical failure, excluding the effects of age and sex, which are known risk factors [8, 11, 13]. This retrospective study demonstrated no significant difference in the surgical success rate of MMC-TLE between the UG and POAG groups. Although the preoperative IOP was higher in UG than in POAG, the number of postoperative antiglaucoma medications to control IOP was slightly lower in UG in the long term.

Surgical outcomes of MMC-TLE for UG in this study (a 66.5% success rate: <18 mmHg and >20% reduction from baseline) were similar to those reported in previous studies involving a relatively large number of cases. Kaburaki et al. [10] reported a 64.7% success rate (≤15 mmHg), Iwao et al. [12] reported a 71.3% success rate (<21 mmHg), Shimizu et al. [13] reported an 82.9% success rate (<21 mmHg), and Bettis et al. [14] reported a 66.7% success rate (<21 mmHg) for MMC-TLE in UG. These previous retrospective studies suggested that surgical outcomes of MMC-TLE for UG are comparable or slightly inferior to those for POAG. In our study, there was no significant difference in the cumulative probability of surgical success between the UG and POAG groups during the long-term observation period (120 months).

Previous studies of MMC-TLE in UG patients also suggested that surgical success depends on postoperative inflammation [9, 10]. In addition, sex (male) [8, 13] and age (less than 30 [11] or 45 years [13]) were reported as risk factors for surgical failure. On the other hand, granulomatous uveitis was reported to be both a risk factor for surgical failure [12] and a factor for surgical success [13].

In this study, exacerbated inflammation postoperatively was found in 6 eyes (12%) out of UG eyes and none in POAG eyes. As in previous reports [10–14], UG eyes sometimes showed postoperative exacerbated inflammation despite receiving preoperative anti-inflammatory treatment unlike POAG eyes. The exacerbated inflammation in UG eyes was significantly correlated with surgical failure at 36 months after surgery, while it was not a significant risk factor for surgical failure at 120 months in this study. In the medium term, ocular inflammation control is important for successful surgery, but in the long term, it might be relatively small as a risk factor.

For granulomatous uveitis, our results supported the aforementioned report by Shimizu et al. [13] because granulomatous uveitis was significantly associated with surgical success at 120 months after surgery. We considered the reason for this to be because our patients with granulomatous uveitis included elderly female sarcoidosis patients. Elderly female sarcoidosis patients with controlled inflammation were less prone to filtration bleb failure than young male patients with nongranulomatous uveitis. Iwao et al. [12] reported that MMC-TLE was less effective at maintaining IOP reduction in UG than in POAG, and granulomatous uveitis was a risk factor for surgical failure. However, their UG group was significantly younger than their POAG group. These differences may have been caused by the age-matching of the UG and POAG groups prior to this study.

In addition, cataract progression after MMC-TLE in UG is an important issue [8, 10, 12]. Although there was no significant difference, there were more patients in the UG group who required cataract surgery within 1 year after surgery than in the POAG group (14.7% and 4.8%, *P*=0.14). Possibly, due to the small sample size, neither combined cataract surgery nor additional cataract surgery within 1 year was a risk factor for surgical failure of MMC-TLE in our study.

In the previous comparative studies between MMC-TLE alone and simultaneous cataract surgery, a prospective study has suggested better outcomes with the single surgery [22], while most of studies showed no statistically significant difference between the two [23–25]. Most of the subjects in these studies were patients with POAG or exfoliation glaucoma, and few patients with secondary glaucoma were included. To the best of our knowledge, there is no report that has examined the difference in efficacy between MMC-TLE alone and simultaneous cataract surgery for UG. The other previous studies have shown that UG eyes which had undergone phacoemulsification after MMC-TLE had a worse prognosis [26] or required more medications to control the IOP [27] than eyes which had not undergone phacoemulsification. Therefore, if cataract progression is expected, MMC-TLE combined with phacoemulsification may also be a surgical option, as previous studies have shown [9, 16].

The limitations of our study were its retrospective nature, the involvement of multiple surgeons, and incomplete standardized surgical procedure (e. g., scleral flap suture and conjunctival suture) and postoperative procedures (e. g., timing of laser suture lysis, ocular massage, and additional antiglaucoma medications). We did not collect quantitative data by laser flare-cell meter with respect to the degree of intraocular inflammation and the subjects had a wide range of uveitis.

## 5. Conclusion

MMC-TLE effectively reduced the IOP in both UG and POAG groups. There was no significant difference in the long-term success rates between UG and POAG. However, postoperatively exacerbated inflammation in UG was significantly correlated with surgical failure at 36 months after surgery, although it was not a significant risk factor for surgical failure at 120 months. Non-granulomatous uveitis was a significant prognostic factor for surgical failure of MMC-TLE in UG at 120 months. If there is sufficient inflammation suppression by medical treatment, surgical outcomes of UG may be comparable with those of POAG in the long term.

## Figures and Tables

**Table 1:** Baseline patient characteristics

	UG (50 eyes)	POAG (50 eyes)	*P*-value
Sex (male/female)	21/29	21/29	1.00
Age (years)	62.4 ± 12.9	65.0 ± 9.4	0.28
Combined with cataract surgery	8	8	1.00
Phakia/pseudophakia or aphakia	34/16	42/8	0.10
Previous suture trabeculotomy	2	1	1.00
Preoperative IOP (mmHg)	27.6 ± 10.6	18.0 ± 4.5	<0.001^*∗∗∗*^
Preoperative antiglaucoma medications (no)	2.9 ± 0.7	3.0 ± 0.4	0.51
Preoperative oral CAIs	30	8	<0.001^*∗∗∗*^
Preoperative MD (dB) in HFA	−16.3 ± 8.3	−19.4 ± 6.5	0.14
Postoperative follow-up period (months)	63.5 ± 30.7	75.8 ± 28.9	0.08

Age: the mean age at the time of surgery (mean ± standard deviation). UG: uveitic glaucoma. POAG: primary open angle glaucoma. IOP: intraocular pressure. CAIs: carbonic anhydrase inhibitors. HFA: Humphrey field analyzer. *P*-value (Student's *t-*test, Fisher's exact test), ^*∗∗∗*^*P* < 0.001

**Table 2:** Uveitis phenotypic diagnosis

Clinical entity	No. of eyes	%
Sarcoidosis	17	34 (%)
Vog*t-*Koyanagi-Harada disease	4	8 (%)
Behçet's disease	4	8 (%)
Varicella zoster virus uveitis	2	4 (%)
Fuchs' heterochromic iridocyclitis	1	2 (%)
Cytomegalovirus uveitis	1	2 (%)
Unknown	21	42 (%)
Total	50	100 (%)

**Table 3:** IOP and antiglaucoma medication after MMC-TLE in UG and POAG

Follow−up (mo)	UG		POAG	
	IOP (mmHg)	Medications	IOP (mmHg)	Medications
1	10.8 ± 4.4	0.0 ± 0.0	11.6 ± 4.7	0.1 ± 0.4
6	11.0 ± 3.8	0.2 ± 0.5^*∗*^	11.5 ± 4.0	0.7 ± 1.0
12	11.7 ± 4.2	0.3 ± 0.8^*∗∗*^	12.2 ± 3.8	0.8 ± 1.1
18	11.2 ± 4.2	0.3 ± 0.7^*∗∗*^	12.2 ± 3.4	1.1 ± 1.2
24	11.0 ± 4.0	0.5 ± 1.1^*∗∗*^	12.1 ± 3.1	1.3 ± 1.3
30	10.8 ± 4.7^*∗*^	0.6 ± 1.1^*∗∗*^	12.5 ± 3.0	1.4 ± 1.3
36	11.9 ± 7.0	0.7 ± 1.2^*∗∗*^	12.1 ± 3.1	1.5 ± 1.3
42	11.4 ± 4.6	0.9 ± 1.4	11.9 ± 3.3	1.5 ± 1.4
48	10.5 ± 4.1	1.1 ± 1.5	11.5 ± 3.2	1.6 ± 1.4
54	10.9 ± 3.9	1.3 ± 3.9	11.9 ± 3.3	1.5 ± 1.4
60	10.6 ± 4.0	1.3 ± 1.7	11.7 ± 3.9	1.6 ± 1.4
66	10.3 ± 3.6	1.6 ± 1.6	12.0 ± 4.4	1.7 ± 1.5
72	10.8 ± 4.8	1.6 ± 1.8	11.0 ± 3.1	1.8 ± 1.6
78	10.0 ± 3.8	1.7 ± 1.7	11.9 ± 4.5	1.7 ± 1.7
84	10.5 ± 3.9	1.1 ± 1.3	11.6 ± 3.8	1.7 ± 1.7
90	10.4 ± 4.3	1.1 ± 1.3	11.5 ± 4.0	1.8 ± 1.8
96	9.2 ± 3.1	0.9 ± 1.1	11.1 ± 3.0	1.9 ± 1.6
102	8.8 ± 2.9	0.6 ± 1.0^*∗*^	11.8 ± 5.4	2.2 ± 1.7
108	10.1 ± 3.7	0.5 ± 1.1^*∗*^	13.1 ± 5.5	2.7 ± 1.6
114	9.7 ± 5.4	0.2 ± 0.4^*∗*^	12.6 ± 6.2	2.4 ± 1.7
120	13.0 ± 5.2	0.2 ± 0.4^*∗*^	10.6 ± 1.2	2.6 ± 1.5

UG: uveitic glaucoma. POAG: primary open angle glaucoma. IOP: intraocular pressure (mean ± standard deviation). “Medications” indicates the number of different antiglaucoma medications applied regardless of their concentration and frequency. (^*∗*^*P* < 0.05, ^*∗∗*^*P* < 0.01 Student's *t-*test).

**Table 4:** Postoperative complications and additional surgeries.

	UG	POAG	*P*-value
Complications			
Aqueous leakage	4 (8.0%)	10 (20.0%)	0.15^*∗*^
Shallow anterior chamber	3 (6.0%)	5 (10.0%)	0.72
Choroidal detachment	7 (14.0%)	2 (4.0%)	0.16
Hyphema	7 (14.0%)	2 (4.0%)	0.16
Exacerbated inflammation	6 (12.0%)	0 (0.0%)	0.03^*∗*^

Additional surgeries			
Additional glaucoma surgery	8 (16.0%)	4 (8.0%)	0.36
Cataract surgery within 12 months	5/34 (14.7%)	2/42 (4.8%)	0.14

^*∗*^ *P* < 0.05, Fisher's exact test. There were 34 phakic eyes in UG and 42 in POAG.

**Table 5:** Multivariate logistic regression analysis of the risk factors for surgical failure at 3 years in UG.

	Odds rate	95% CI	*P*-value
Men	0.63	0.07−5.53	0.68
Age	0.95	0.88−1.03	0.20
Granulomatous uveitis	0.12	0.01−1.80	0.12
Panuveitis	0.27	0.03−2.21	0.22
Combined with cataract surgery	0.25	0.01−7.51	0.42
Pseudophekic eye	0.54	0.04 −7.63	0.65
Preoperative IOP	0.99	0.91−1.08	0.87
Postoperatively exacerbated inflammation	74.4	1.83−3029.03	0.02^*∗*^
Postoperative cataract surgery	0.47	0.03−6.74	0.58

CI: confidence interval. ^*∗*^*P* < 0.05.

**Table 6:** Multivariate logistic regression analysis of the risk factors for surgical failure at 10 years in UG

	Odds rate	95% CI	*P*-value
Men	1.60	0.32−8.14	0.57
Age	0.97	0.92−1.03	0.32
Granulomatous uveitis	0.14	0.02−0.92	0.04^*∗*^
Panuveitis	1.52	0.22−10.60	0.67
Combined with cataract surgery	0.28	0.015−5.47	0.40
Pseudophekic eye	0.25	0.02−2.65	0.25
Preoperative IOP	1.00	0.93−1.08	0.94
Postoperatively exacerbated inflammation	10.83	0.51−229.15	0.13
Postoperative cataract surgery	1.78	0.26 −12.33	0.56

CI: confidence interval. ^*∗*^*P* < 0.05.

## Data Availability

The data used to support the findings of this study are available from the corresponding author upon request.
